# Is intracranial volume a suitable proxy for brain reserve?

**DOI:** 10.1186/s13195-018-0408-5

**Published:** 2018-09-11

**Authors:** Anna Catharina van Loenhoud, Colin Groot, Jacob William Vogel, Wiesje Maria van der Flier, Rik Ossenkoppele

**Affiliations:** 1 Department of Neurology and Alzheimer Center, Neuroscience Campus Amsterdam, VU University Medical Center, Amsterdam, The Netherlands; 2 Montreal Neurological Institute, McGill University, Montreal, QC Canada; 3 Department of Radiology and Nuclear Medicine, Neuroscience Campus Amsterdam, VU University Medical Center, Amsterdam, The Netherlands; 4 Department of Epidemiology and Biostatistics, VU University Medical Center, Amsterdam, The Netherlands; 5 Department of Clinical Memory Research, Lund University, Lund, Sweden

**Keywords:** Brain reserve, Intracranial volume, Alzheimer’s disease, Dementia, MRI, Resilience

## Abstract

Brain reserve is a concept introduced to explain why Alzheimer’s disease (AD) patients with a greater brain volume prior to onset of pathology generally have better clinical outcomes. In this review, we provide a historical background of the emergence of brain reserve and discuss several aspects that need further clarification, including the dynamic or static nature of the concept and its underlying mechanisms and clinical effect. We then describe how brain reserve has been operationalized over the years, and critically evaluate the use of intracranial volume (ICV) as the most widely used proxy for brain reserve. Furthermore, we perform a meta-analysis showing that ICV is associated with higher cognitive performance after adjusting for the presence and amount of pathology. Although we acknowledge its imperfections, we conclude that the use of ICV as a proxy for brain reserve is currently warranted. However, further development of more optimal measures of brain reserve as well as a more clearly defined theoretical framework is essential.

## Background

**Fig. 1:**
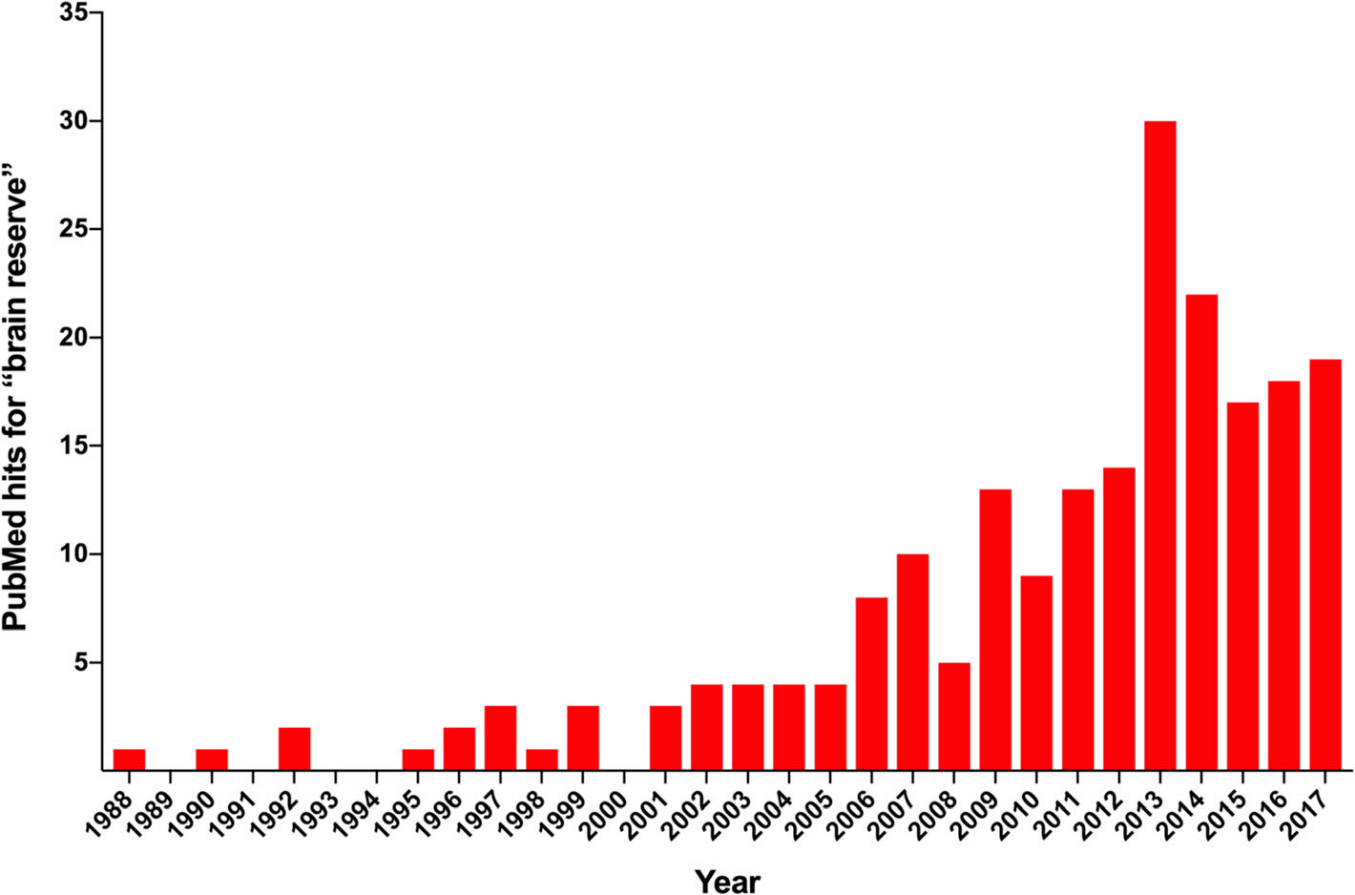
An overview of the yearly number of publications on brain reserve. Search query: “brain reserve” (exact match). No additional filters were applied

The concept of “brain reserve capacity” finds its origins in the scientific literature in the first half of the twentieth century. An article in 1940 described the remarkable observation of a 27-year-old post-traumatic epilepsy patient who retained a relatively normal intellect and personality despite surgical removal of large parts of his brain. This maintenance of function after surgery was also reported in monkeys and rats and was especially apparent when performed at a relatively young age [1]. These studies demonstrate the capacity of the brain to utilize remaining (or reserve) brain tissue to take over functions from brain regions and networks affected by injury. Approximately 40 years later, Roth [49] described a similar phenomenon in the context of dementia. He noted that many neurodegenerative diseases seem to have a long-lasting preclinical phase in which brain pathology is present without the co-occurrence of clinical symptoms. In Alzheimer’s disease (AD), for example, there is now compelling evidence that amyloid-β and tau pathology accumulate decades prior to the onset of cognitive impairment [11, 27, 28]. In his “threshold model of dementia”, Roth argued that during this preclinical phase a protective mechanism of the brain must be responsible for counteracting the effects of pathology until the pathology increases to a critical threshold at which clinical manifestation cannot be prevented any more [49]. This idea was further developed by Mortimer [42] and later also by Satz [52], who added an important dimension to the model by proposing that this pathological threshold is not uniform across individuals: some people need more pathology than others for clinical symptoms to arise. A person who initially has a larger and better-connected brain (i.e., higher premorbid brain reserve) will have more functional brain tissue remaining at a given level of pathology and will thus develop clinical symptoms at a more advanced biological stage. In other words, according to these authors, it is not the amount of pathology per se but its effect on the level of brain reserve that determines whether and when clinical manifestations occur. Their models thus concern a “reserve threshold” rather than a “pathological threshold” [42, 52]. Another influential study that provided additional evidence that pathological thresholds for clinical expression vary between individuals was a postmortem examination described by Katzman et al. [31]. These authors described a group of subjects who showed marked presence of amyloid-β plaques and neurofibrillary tangles (and therefore met the neuropathological criteria for AD) but who had expressed minimal clinical symptoms during life. Further analyses revealed that the brains of these individuals were characterized by a higher weight and a greater number of neurons. These results led the authors to hypothesize that a larger brain size may be protective against the clinical expression of pathology “through the mechanism of reserve” [31]. Years later, Stern [60] further refined the definition of brain reserve by discerning it from cognitive reserve. While often used interchangeably in the past, Stern proposed that brain reserve is a “passive” concept (see glossary) that can be defined by the plain quantity of neural resources supporting the brain to better tolerate emerging neuropathology. In contrast, cognitive reserve, a related but distinct concept that will not be further discussed in this paper, should be considered as an “active” phenomenon (see glossary), referring to the capacity of the brain to cope with damage through more efficient use of pre-existing neural pathways or via recruitment of alternative brain networks [60]. After its introduction in 1940 and continued development throughout the following decades, the concept of brain reserve has been increasingly used in the literature (Fig. 1).

## Unclarified theoretical issues

Despite the significant efforts described above, there is currently no clear consensus on several aspects of the definition and theory behind brain reserve. While there is some consensus among experts in the field that brain reserve describes the phenomenon in which a larger brain size protects against the clinical consequences of pathology, many details remain to be clarified. In the sections below, we will consider issues regarding the dynamic or static nature (see glossary) of the concept and evaluate different theoretical models to explain the mechanisms and clinical effects of brain reserve.

### Dynamic or static nature of brain reserve

An issue that is currently unresolved is a lack of agreement on whether brain reserve is a dynamic or a static concept. Brain size is largely determined by biological and genetic influences [5, 48]. Since intracranial volume (ICV; discussed in more detail below, also see glossary) does not increase after the age of 10 years [47] and the brain has reached maturity around 25 years of age [20, 34, 35], brain reserve was initially thought of as a fixed concept. However, recent literature also emphasizes the role of environmental factors in dynamically shaping brain reserve over the course of life (e.g., [4]). While neuroscientists are still debating whether neurogenesis actually occurs in the adult human brain [57], it is generally accepted that in specific areas (e.g., the hippocampus and subventricular zone) new neurons (and synapses) are formed throughout life [7, 16, 32, 59]. This process is regulated by several lifestyle factors, such as exercise, diet, and social interactions [33, 66]. Brain reserve could therefore potentially increase over time and may thus be more dynamic than originally assumed.

**Fig. 2:**
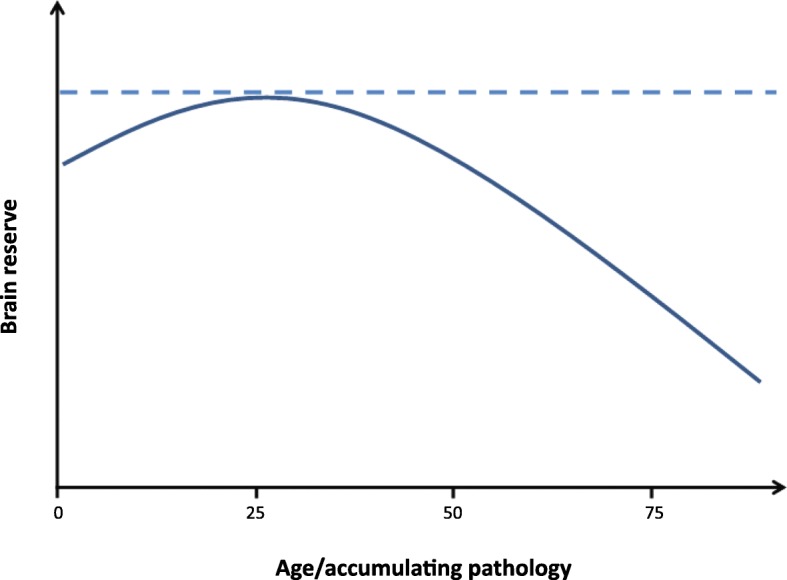
Two (competing) conceptualizations of brain reserve. While some researchers define the concept as the maximum attained volume during life (**a**), others regard it as a more dynamic construct that reflects current brain status, which changes as a function of aging and accumulation of pathology (**b**)

A related theoretical debate concerns whether brain reserve decreases over time as a function of chronological aging or accumulating pathology. While some researchers conceptualize brain reserve as the maximum attained volume during life (e.g., “static” [70]), others have referred to it as the status of the brain at any point in time (e.g., “dynamic” [61]). To illustrate the difference between these interpretations, we take the hypothetical example of an individual who develops late-onset AD. Before accumulation of AD pathology (i.e., amyloid plaques and neurofibrillary tangles), the brain has undergone other pathological changes (e.g., aggregation of other misfolding proteins and white matter lesions) and volume loss as a function of chronological aging. According to the first interpretation, this individual’s brain reserve is the volume of his brain prior to the onset of any age- or disease-related changes. Regardless of the volumetric decreases that occur after that point, his brain reserve (i.e., his maximum attained brain size) will remain the same. In contrast, the second conceptualization of brain reserve depends on which point of time is considered; it will be considerably lower at 80 years of age compared with a younger age, when there is a paucity of comorbid pathologies. See Fig. 2 for a schematic representation of both conceptualizations of brain reserve.

### Mechanisms behind brain reserve and effects on clinical progression

**Fig. 3:**
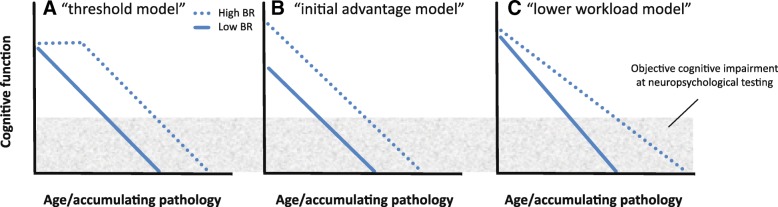
Three possible models of the effects of brain reserve (BR) on clinical progression. **a** The “threshold model”: accumulation of pathology initially has no clinical effect in individuals with higher BR, and only results in cognitive decline after a certain inflection point. **b** The “initial advantage model”: higher BR is associated with a higher premorbid level of cognitive function, and thus more cognitive decline is needed before an objective level of cognitive impairment is reached. **c** The “lower workload model”: higher BR places less workload on individual neurons, and thus the loss of structure leads to relatively little cognitive decline

There is very limited literature on the mechanisms underlying brain reserve and its specific effect on clinical progression. It has been suggested that greater brain reserve (e.g., higher synaptic density, neuronal count, or even a higher glia-to-neuron ratio) optimizes “the potential for functional adaptation to neurodegenerative processes” [53]. In this sense, the mechanism of brain reserve seems to be nothing more than “allowing more cognitive reserve”. Another, more generally used statement that is reminiscent of Mortimer and Satz’s theoretical models, is that higher brain reserve concerns “a higher (pathological) threshold before clinical symptoms of pathology become evident” (e.g., [64]). This description is rather abstract in the sense that the biological processes underlying this “threshold effect” are not specified. To allow advances in the field of brain reserve, it is essential to develop a mechanistic model explaining the link between greater brain volume and a higher threshold for clinical expression of pathology. The term “threshold” suggests that passive loss of brain structure without functional adjustment (which would reflect cognitive reserve) could occur in the absence of any cognitive effects, at least in the initial stages of AD (Fig. 3a, the “threshold model”). However, this implies that the affected structural properties (e.g., neurons, axons, synapses) were fully redundant and served no function in the healthy brain. With the exception of apoptosis (i.e., a coordinated death of cells that no longer contribute to healthy functioning, which is crucial for normal brain development [26]), this is unlikely from a biological perspective. A more likely scenario is that brain reserve is primarily associated with individual differences in premorbid levels of cognitive function, such that individuals with larger brains must undergo greater decrements in cognitive function before a level of objective clinical impairment is reached (Fig. 3b, the “initial advantage model”). In line with this hypothesis, current literature shows evidence of a direct relationship between brain size and general mental ability in cognitively normal adults [51].

Another possibility is that brain reserve is not (only) associated with higher premorbid cognitive function or a delayed cognitive decline, but rather a slower rate of clinical progression. According to Lövdén et al., the brain’s level of cognitive function is a result of the interplay between environmental demands and functional organismic supply [36]. This suggests that, although larger brains have the potential for a higher level of cognitive function, an individual’s actual premorbid level is determined by the cognitive complexity that is required for daily activities. Based on the premise that i) cognitive ability depends on environmental demands and ii) a healthy brain does not maintain functionally obsolete structural properties, a large brain would have more “computational units” available to achieve a given cognitive level than a smaller brain. Consequently, the workload placed on each individual neuron should be lower, resulting in a relatively small decrease in function with the loss of any particular structure (Fig. 3c, the “lower workload model”). Figure 3 depicts the various proposed mechanisms of brain reserve and their clinical effects (note that Fig. 3b and c are similar to the “further to fall” and “resistance to change” models from [53]). Future research should examine which model best captures the effects of a larger brain volume on clinical progression in the face of pathology.

## Operationalization of brain reserve

While further debate on several theoretical aspects of brain reserve is thus needed, many practical attempts to find a suitable proxy for this concept have been made. The notion of brain reserve as a passive model that concerns the “hardware” (i.e., structural, anatomical; see glossary) rather than the “software” of the brain (i.e., functional processes; see glossary) has important implications for the operationalization of this concept. It has led to the assumption that brain reserve should be measured in a quantitative manner, for example by the number of neurons, synapses, and/or dendritic spines [10]. An attractive aspect of this hypothesis is that it does not attribute any specific neuronal feature or combination of features as the mediating substrate of reserve, and that it is differentiable from larger concepts of cognitive networks that might underlie cognitive reserve. Whatever the substrate of brain reserve may be, bigger brains allow for more of it, and more of it is better in the face of pathology, or so the hypothesis would state. This phenomenon would be represented as a net advantage rather than an overall advantage, as there are likely some (e.g., metabolic) costs to the maintenance of a larger brain [43]. Prior to the advent of brain imaging techniques, head circumference was often used as an in-vivo measure of brain size to quantify brain reserve. The rationale behind this is that the ultimate size of the cranial vault is determined by internal pressure from expanding brain parenchyma [62], and thus head circumference reflects the maximum attained brain size. There is evidence suggesting that head circumference indeed captures the influence of brain reserve on clinical outcomes. For example, a study including ~ 2000 older individuals showed that persons with probable AD and a circumference below 55 cm performed significantly worse on neuropsychological testing [21]. Another longitudinal study revealed that, among persons without dementia who carried the major risk allele for sporadic AD (apolipoprotein E ε4), head circumference was related to future development of AD dementia [8]. Nowadays, brain size can be more precisely estimated using T1-weighted structural magnetic resonance imaging (MRI) scans. This estimation can be performed manually or based on an automated process that either i) determines the scaling factor or nonlinear transformation needed to register a native space image to an average template, or ii) calculates the total sum of probabilistic tissue class images (i.e., gray matter + white matter + cerebrospinal fluid volumes) [12, 39]. The intracranial volume (ICV) measure resulting from these techniques is currently the most accepted operationalization of brain reserve.

## A critical appraisal of intracranial volume as a brain reserve proxy

ICV is an easily accessible measurement to summarize variation in premorbid brain size and is an attractive proxy for brain reserve because it represents an absolute limit on individual brain volumetric capacity. However, using ICV as a proxy for brain reserve comes with practical issues. First, ICV is not necessarily a straightforward measurement of total brain capacity as cortical surface area varies (in the form of folding) with some independence to brain size in humans [37, 63]. This implies that more subtle individual differences in brain reserve may not be captured by a volumetric measure such as ICV. It should also be stated that, while automated estimations of ICV show excellent reliability with human raters [39], variations in these automated approaches still tend to be systematically biased by other confounders, such as gender and total brain atrophy [45]. Another disadvantage of using ICV as a brain reserve proxy is that, in healthy individuals, brain volume varies systematically with ICV, leading to near uniform recommendation of ICV as a nuisance covariate in volumetric studies [6, 13, 25, 55]. Whether this confounding relationship is caused by the natural influence of brain volume on cortical shape [63], systematic error in linear registration [54], or a combination is unclear. Regardless, the question must be raised whether a measurement can be both a proxy for brain reserve and a common nuisance covariate, and how this might play out in the context of neuroimaging studies. Little work has been done to examine or disentangle the methodological versus biological influence of ICV on morphometry, or how either may somehow relate to cognitive outcomes. In effect, covarying a morphometric analysis for ICV is both adjusting for the effects of linear transformation to a common space (a necessary step), but also incorporating information about premorbid brain state into the model. This somewhat convoluted concept remains a limitation of using ICV as a proxy for brain reserve, particularly in volumetric studies of aging and AD. Its confounding relationship with brain morphometry creates difficulties in planning a clean design of neuroimaging studies of the brain (and cognitive) reserve (e.g., [68]). Finally, the more dynamically we define brain reserve on a conceptual level (see previous section on unclarified theoretical issues), the more indirect becomes its relationship with ICV. As ICV is a fixed measure that reflects maximum attained brain volume, it does not change as a function of chronological aging or emergence of a neurodegenerative disease [24]. As a consequence, both positive and negative changes in brain reserve (e.g., due to lifestyle, or aging and pathology) cannot be captured with this proxy.

## Meta-analysis of the effect of intracranial volume on cognition in Alzheimer’s disease

So far, our narrative review of the literature suggests a possible beneficial effect of ICV as a proxy for brain reserve on cognitive function. Results, however, have been mixed across studies, with some studies indicating a positive effect [22] while others report no effect [58] or even a negative effect [15]. We set out to systematically review the available literature quantifying the effects of ICV on cognitive function, and aggregate all results into a meta-analysis. Since the primary focus of this viewpoint is on aging and AD, only studies including these populations were included in the meta-analysis. Please note that, although we raise several critical points on a theoretical level regarding the use of ICV as a brain reserve proxy, we nevertheless aim to examine its empirical usefulness because it is currently the most accepted and widely used measure of brain reserve.

### Methods

We searched the PubMed/MEDLINE database for eligible studies published until 8 November 2017. The following combination of search terms were applied: “(Intra(−)cranial volume(s)/capacity/size/space”, “(pre(-)morbid) brain size/volume”, “dementia”, “AD”, “mild cognitive impairment/MCI”, “elderly”, “ag(e)ing” and “(cognitive/brain/neural) reserve”. Additionally, reference lists of papers were cross-checked. Only peer-reviewed articles (written in or translated to English) were considered eligible. Studies were required to include a continuous or categorical (e.g., small versus large volume) measure of ICV as measured by MRI. ICV could be assessed either as a main predictor or a covariate (if an effect size was reported). Furthermore, samples could include the cognitively normal elderly, or patients with mild cognitive impairment (MCI) or dementia [3, 40]. Studies exclusively including patients with neurodegenerative diseases other than AD were excluded. Samples including patients with mixed or unknown dementia diagnoses were included (since AD is the most likely a priori diagnosis), as well as samples combining AD patients with a small proportion of patients with an alternative diagnosis. Predicted measures should contain a clinical outcome, either continuous (e.g., Mini-Mental State Examination (MMSE) or episodic memory scores) or categorical (e.g., cognitively impaired (yes/no) or longitudinal conversion to cognitive impairment). Importantly, since brain reserve serves to explain discrepancies between observed and expected symptom severity based on the severity of underlying pathology [30], studies were required to include an operationalization of neuropathology (e.g., atrophy; see glossary). In order to examine the effects of ICV on cognition at a given level of pathology, studies should have included this operationalization of neuropathology in the same model as ICV. OpenMetaAnalyst software was implemented to determine the overall effects of ICV on quantified measures of cognition. Due to expected heterogeneity (e.g., sample composition and nuisance variables), effects sizes were assumed to be similar but not equal across studies. Therefore, a random-effects meta-analysis was performed [9]. Significance for random effects of ICV was set at *p* < 0.05.

### Results

#### Study description

**Fig. 4:**
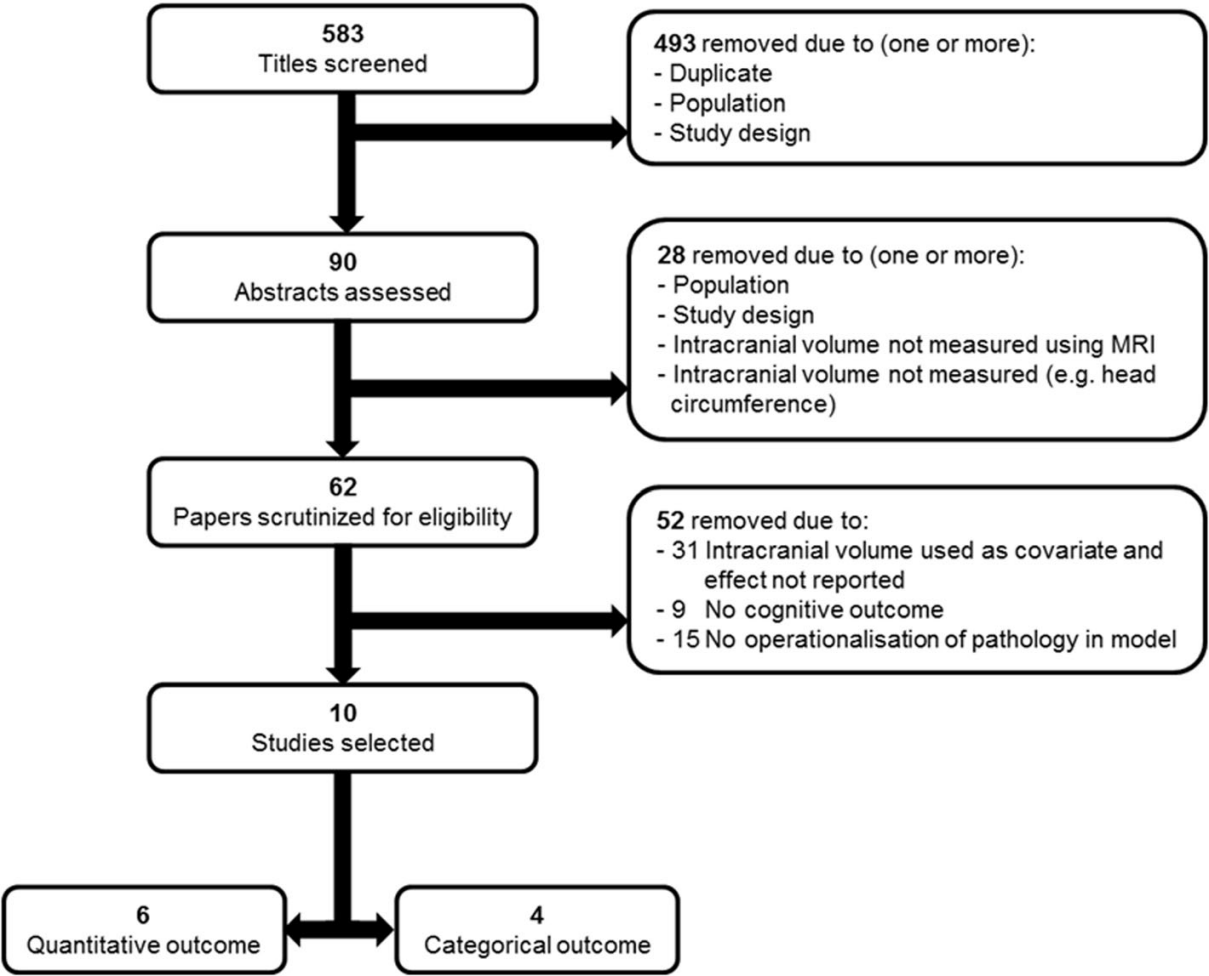
Flow diagram depicting study selection. MRI magnetic resonance imaging

Database queries yielded a total of 583 results, of which 10 were eligible to be included (Fig. 4) [15, 18, 22, 41, 44, 50, 56, 58, 69, 70].

These 10 studies included a total of 2675 patients. Two studies [69, 70] likely included a partly overlapping sample of subjects since these study samples were taken from the same cohort. Two studies included (sub)samples of only AD dementia patients [22, 41] and one included only healthy elderly [50]; all other samples were mixed (e.g., healthy elderly, MCI, and/or dementia subjects) or community samples with unknown diagnoses. The mean age of all subjects was 69.8 ± 5.8 years, 48% were male, and mean education was 11.8 ± 1.3 years. All studies calculated ICV by summation of gray matter, white matter, and cerebrospinal fluid volumes, except for [41] which defined premorbid brain volume by regressing ICV on whole brain volume, while correcting for age and sex. To obtain the premorbid brain volume, the regression coefficient was multiplied by the ICV and the constant was added. Across studies, different nuisance variables were taken into account, but most models included age and sex (Table 1). Furthermore, operationalization of neuropathology included in the models also varied across studies but could be roughly divided into measures of hippocampal volume, white matter, gray matter, and total brain volume (Table 1).

**Table 1:** Study characteristics

Study	*n*	Subjects	Age	Male (%)	Education (years)	Design	Nuisance	Corrected for	Outcome	Effect
Quantitative assessment										
Mori, 1997* [41]	60	Mild to moderate AD	70.2 (7.1)	38.3	8.9 (2.3)	Cross-sectional	Age, sex, education	Atrophy	ADAS-Cog^a^	−0.12
									WAIS-R Full IQ^e^	0.40
Staff, 2004 [58]	98	Unknown	79	57.6	9.8 (1.6)	Cross-sectional	Childhood IQ, sex	WMH	AVLT Memory^b^	0.00
									RPM^e^	0.01
Christensen, 2009 [15]	416	Unknown	62.6 (1.4)	52	14 (2.6)	Longitudinal, 4-year change	Age, sex, education	Atrophy and WMH	SDMT^c^	−0.22
									CVLT: Immediate^b^	−0.39
									CVLT: Delayed^b^	**−1.45** ^**†**^
Farias, 2012 [18]	401	Mixed HC, MCI and dementia	75 (6.9)	37.3	12.3 (4.8)	Cross-sectional	Sex, height	TBV, hippocampal volume, and WMH	SENAS: Semantic memory^b^	**0.20**
									Episodic memory^b^	0.08
									Executive function^c^	**0.27**
									Spatial ability^d^	**0.16**
Royle, 2013 [50]	327	HC	72.5 (0.7)	100	–	Cross-sectional	Age	TBV	Composite score^a^	**0.19**
								GM and WM		**0.22**
	297		72.6 (0.73)	0	–			TBV		**0.21**
								GM and WM		**0.23**
Groot, 2018 [22]	201	Aβ+ preclinical and prodromal AD	66.6 (7.5)	53	10–11**	Cross-sectional	Age, sex, education, scanner	Atrophy	Memory^b^	0.12
									Attention^c^	0.06
									Executive^c^	**0.18**
									Language^f^	−0.03
									Visuospatial^d^	0.14
									MMSE^a^	0.16
	462	Aβ+ probable AD	66.1 (7.4)	47	10–11**				Memory^b^	0.10
									Attention^c^	**0.14**
									Executive^c^	**0.15**
									Language^f^	0.05
									Visuospatial^d^	**0.13**
									MMSE^a^	**0.15**
Categorical assessment										
Wolf, 2004 [69]	73	HC, MCI	79.1	49.3	11.3	Cross-sectional	Education	Left hippocampus	Predicting HC vs MCI (OR)	1.04***
	70	MCI, dementia	78.7	34.3	10.8		Age	RBV	Predicting MCI vs dementia (OR)	1.05***
Wolf, 2004 [70]	167	HC, MCI, AD, VaD	60.7 (9.9)	43	–	Cross-sectional	Age, sex	Hippocampal atrophy (visual assessment)	HC vs cognitive impairment (OR compared with smallest quartile)	**2.9**
Silbert, 2009 [56]	49	HC (at baseline)	84.1 (6.2)	47	14.5 (2.7)	Longitudinal, 10-year change	Age, MMSE, APOEe4 status.	ΔWMH, ΔvCSF, and hippocampal, vCSF and WMH	Persistent cognitive decline (HR)	1.0
Negash, 2013 [44]	54	Aβ+ HC and AD	72.7	42.6	14.4	Cross-sectional	Age, sex, education, APOEe4 status	MTL volume	Resilience (normal despite Aβ+; OR)	**1.01**
								Hippocampal and posterior cingulate volume		**1.01**

Bold effects are reported to be significant according to study-specific statistical thresholds

Aβ amyloid-beta, AD Alzheimer’s disease, ADAS-Cog Amsterdam dementia assessment scale—cognitive subscale, APOEε4 apolipoprotein ε4, AVLT auditory verbal learning test, CVLT California verbal learning test, GM gray matter, HC healthy controls, HR hazard ratio, ICV intracranial volume, IQ intelligence quotient, MCI mild cognitive impairment, MMSE Mini-Mental State Examination, MTL medial temporal lobe, OR odds ratio, RBV relative brain volume (brain volume to ICV ratio), RPM Raven’s progressive matrices, SDMT symbol-digit modalities test, SENAS Spanish-English neuropsychological assessment scale, TBV total brain volume, VaD vascular dementia, vCSF ventricular cerebrospinal fluid, WAIS-R Wechsler adult intelligence scale-revised, WM white matter, WMH white matter hyperintensity

*Premorbid brain volume calculated as regression coefficient of (age + sex + ICV = whole brain volume) multiplied by ICV + constant

**Categorization according to the Verhage scale [65] converted into years

***Odds ratios calculated from β coefficients using e^(β)

^†^This effect was considered an outlier and was not included in the meta-analysis

^a^Global cognition

^b^Memory

^c^Attention/executive functions

^d^Visuospatial ability

^e^Intelligence

^f^Language

#### Effects of intracranial volume on cognition

**Fig. 5:**
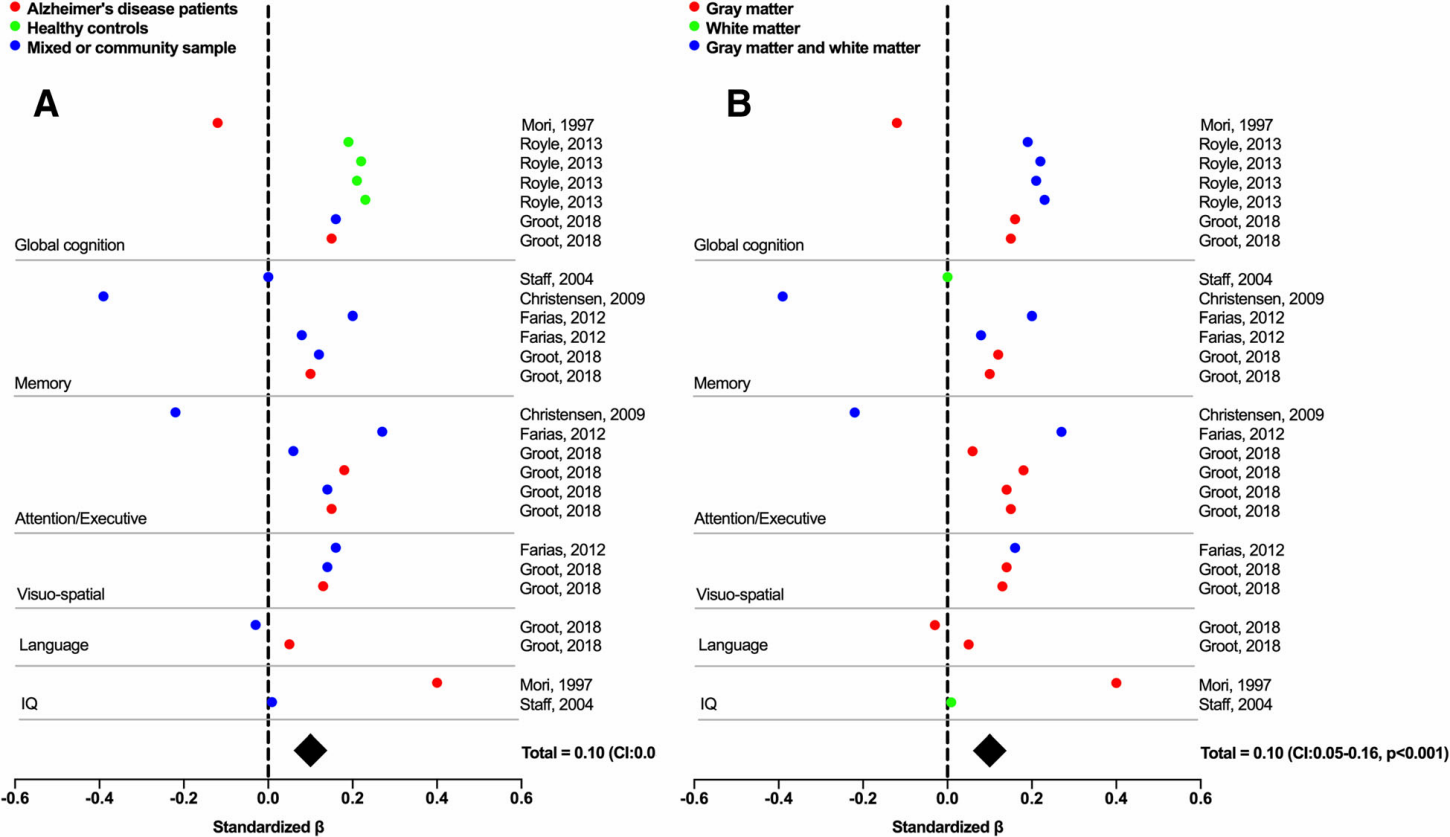
Forest plot of main analysis on quantitative cognitive outcomes. **a** Results according to the cognitive state of the samples; **b** results according to the measure of corrected neuropathology. Data points indicate a reported effect. Standard errors are not displayed as these were rarely reported. Total effect was calculated using random-effects meta-analysis including all effects across cognitive outcomes and populations. CI confidence interval, IQ intelligence quotient

Of the 10 selected studies, six provided a continuous measure of cognition and were therefore suitable to be included in a meta-analysis. Five of these studies had a cross-sectional design and one had a longitudinal design (Table 1). This subsample consisted of 2262 subjects, of which 48.5% were male, the mean age was 69.8 ± 4.9 years, and mean education was 11.7 ± 1.2 years. Across these six studies, 26 cognitive tests were performed which could be categorized into the following domains: global cognition, memory, attention and/or executive functions, visuo-spatial ability, language, and IQ. An overview of each test used and the categorization into domains is provided in Table 1. One study [15] reported an effect of −1.45 of ICV on the California verbal learning test, delayed recall condition, which was a statistical outlier and therefore excluded from the analyses. The main analysis for the quantitative assessments of cognition, including all cognitive domains, revealed a positive random effect of ICV on cognition (0.10, 95% confidence interval (CI) 0.05–0.16; *p* < 0.001; Fig. 5). This indicates that, across all studies, ICV has a positive effect on cognitive functioning when controlling for neuropathology.

Four studies provided categorical cognitive outcomes. A longitudinal assessment [56] reported a hazard ratio of 1.0 for ICV on conversion to persistent cognitive decline, indicating that ICV does not affect the risk of conversion. Another study [69] reported that ICV predicts being cognitively normal versus MCI (odds ratio 1.4) and having MCI versus dementia (odds ratio 1.5), while a similar assessment [70] showed that having an ICV in the lowest quartile confers an odds ratio of 2.9 of being cognitively impaired. Furthermore, another study [44] showed that ICV is a significant covariate (odds ratio 1.01) associated with resilience, defined as being cognitively intact despite positive biomarkers for amyloid-β. Taken together, these results show that, while odds ratio were generally close to 1 (except in [70]), ICV is a significant predictor for cross-sectional cognitive state.

#### Methodological considerations

In this meta-analysis, studies that assessed the effects of ICV on cognition but did not include a measure of neuropathology were not included. The three largest studies that assessed the effects of ICV in this manner found no associations with cognition or dementia risk [17, 29, 62]. However, brain reserve serves to explain discrepancies between observed and expected symptom severity based on the severity of the underlying pathology [30] and we argue that studies that do not correct for the degree of neuropathology do not measure brain reserve. Based on the absence of an effect when not correcting for neuropathology and the presence of an effect when models are corrected for neuropathology, we hypothesize that direct effects of ICV on cognition are not detectable but the “buffer” effect of ICV between pathology and symptoms (which constitutes brain reserve) is detectable.

We would have ideally conducted additional meta-analyses in subsamples of studies that included patients in the same cognitive state (e.g., healthy controls versus MCI versus dementia) and studies using similar measures of neuropathology. However, due to the paucity of studies that fit the inclusion criteria (*n* = 6 for quantitative assessment) this would have resulted in very few studies per analysis. The effects in healthy controls would, for instance, be based on only one study [50] and the differences in effects between healthy subjects and other cognitive states would thus not be distinguishable from study effects. Visual inspection of the effects (color-coded by cognitive state in Fig. 5a) indicates that there are no clearly identifiable differences in effects of brain reserve, but this observation needs to be interpreted with caution. Furthermore, visual inspection of the effects when color-coded according to the corrected marker of neuropathology (Fig. 5b) suggests that there is no effect of ICV when correcting for white matter hyperintensity (WMH) alone. However, this observation is based on results in two samples from a single study [58] and may thus represent a study effect. Finally, we were unable to account for the influence of the different covariate sets used across studies. The most important covariates, age and sex, were included in most—but not all—studies, and adjustment for education, scanner, and *APOE*ε4 status varied across studies. One study also corrected for childhood IQ [58], which may have led to an attenuation of the observed effect in this study due to the association between childhood IQ and ICV [67]. Similarly, one study corrected for height [18]. However, although height and ICV are clearly related [38] and associations between height and cognition have also been found [2] it is possible that correcting for height results in removal or attenuation of true effects of ICV (i.e., brain reserve).

## Conclusion

To summarize, the concept of brain reserve has been around since 1940 and its use in the scientific literature has increased ever since. While consensus on some theoretical aspects of its definition and underlying mechanisms has not yet been reached, the field has made significant progress in the operationalization of brain reserve. ICV, currently the most accepted proxy, is an easily accessible measure obtained from structural MRI. However, it has a limited utility in detailing the biological substrate of brain reserve, and a confounding relationship with brain morphometry that complicates the measurement of brain reserve in volumetric studies. Nonetheless, a meta-analysis of 10 studies showed that ICV generally has a positive relationship with cognitive performance after adjusting for pathology, indicating that this measure does capture some aspect of brain reserve. Although the use of ICV as a proxy for this concept is thus currently warranted, we emphasize the need for further development of more optimal measures of brain reserve. For example, the utility of dynamic measures as proxies of brain reserve (e.g., whole-brain or hippocampal volumes instead of ICV) could be explored, for example using a meta-analysis including studies focusing on associations between absolute volumetric measures (not adjusted for ICV) and cognition. Also, possible candidates in future studies would include diffusion tensor imaging or indices of microstructural integrity of the brain, such as in-vivo examination of dendritic spine length, synaptic density, or synaptic proteins using synaptic vesicle tracers for positron emission tomography (PET) [10, 19]. Another possible avenue for future research is the assessment of the associations between genetic factors and brain reserve. It has already been shown that there is an overlap in the genetic variations associated with cognition and ICV [2, 46]. Another interesting target is the methionine (Met) substitution for valine (Val) at codon 66 (Val66Met), a single-nucleotide polymorphism in the brain-derived neurotrophic factor (BDNF) gene that has been associated with alterations in brain anatomy [14]. Now, with the introduction of the Allen human brain atlas [23], one may explore the interplay between more dynamic measures of brain reserve and the relative gene expression across the entire human brain. These new developments allow exploration of gene expression pathways that contribute to, or mediate the effects of, brain reserve. Ultimately, this could enable an operationalization of brain reserve based on genetic information. Finally, in order to further improve the measurement of brain reserve, a more clearly defined theoretical framework of this concept is essential.

## Abbreviations

AD: Alzheimer’s disease
ICV: Intracranial volume
MCI: Mild cognitive impairment
MRI: Magnetic resonance imaging

## Glossary

Brain volume: The total volume of the brain when all structural properties (i.e., gray matter and white matter) are considered.
Intracranial volume: The total volume of the interior of the cranium (i.e., skull), thus containing both brain volume and cerebrospinal fluid.
Atrophy: Loss of brain structure (e.g., neurons, glial cells).
Passive: A change in the brain to which no direct adaptation or response occurs, or a latent phenomenon or quality that covertly existed but now becomes visible when its surroundings are changing.
Active: A change in the brain to which a direct adaptation or response occurs, or a phenomenon or quality that was born out of necessity to cope with brain changes (antonym of “passive”).
Hardware: The (infra)structure of the brain, which can be measured with structural neuroimaging (in vivo) or at neuropathological examination (ex vivo).
Software: The manner in which the (infrastructure) of the brain is used, which can only be measured with functional neuroimaging during life.
Static: A concept or measure that is fixed; it cannot change over time and thus an individual will always have the same value or score.
Dynamic: A concept or measure that can change over time, such that its values or scores can fluctuate (i.e., increase and decrease) within individuals.
